# MiR‐139‐5p, miR‐940 and miR‐193a‐5p inhibit the growth of hepatocellular carcinoma by targeting *SPOCK1*

**DOI:** 10.1111/jcmm.14121

**Published:** 2019-02-01

**Authors:** Peng Li, Zhiwei Xiao, Jiajun Luo, Yaojun Zhang, Lizhu Lin

**Affiliations:** ^1^ Cancer Center, The First Affiliated Hospital of Guangzhou University of Chinese Medicine Guangzhou Guangdong China; ^2^ Department of Hepatobiliary Surgery Sun Yat‐sen University Cancer Center Guangzhou Guangdong China

**Keywords:** hepatocellular carcinoma, miR‐139‐5p, miR‐193a‐5p, miR‐940, *SPOCK1*

## Abstract

The study was aimed to screen out miRNAs with differential expression in hepatocellular carcinoma (HCC), and to explore the influence of the expressions of these miRNAs and their target gene on HCC cell proliferation, invasion and apoptosis. MiRNAs with differential expression in HCC were screened out by microarray analysis. The common target gene of these miRNAs (miR‐139‐5p, miR‐940 and miR‐193a‐5p) was screened out by analysing the target genes profile (acquired from Targetscan) of the three miRNAs. Expression levels of miRNAs and *SPOCK1* were determined by quantitative real time polymerase chain reaction (qRT‐PCR). The target relationships were verified by dual luciferase reporter gene assay and RNA pull‐down assay. Through 3‐(4,5‐dimethyl‐2‐thiazolyl)‐2,5‐diphenyl‐2‐H‐tetrazolium bromide,thiazolyl blue tetrazolium bromide (MTT) and transwell assays and flow cytometry, HCC cell viability, invasion and apoptosis were determined. In vivo experiment was conducted in nude mice to investigate the influence of three miRNAs on tumour growth. Down‐regulation of miR‐139‐5p, miR‐940 and miR‐193a‐5p was found in HCC. Overexpression of these miRNAs suppressed HCC cell viability and invasion, promoted apoptosis and inhibited tumour growth. *SPOCK1*, the common target gene of miR‐139‐5p, miR‐940 and miR‐193a‐5p, was overexpressed in HCC. *SPOCK1* overexpression promoted proliferation and invasion, and restrained apoptosis of HCC cells. MiR‐139‐5p, miR‐940 and miR‐193a‐5p inhibited HCC development through targeting *SPOCK1*.

## 1 INTRODUCTION

Hepatocellular carcinoma (HCC), a primary liver cancer, is characterized with poor prognosis and high mortality.^1^ The 5‐year survival rate for HCC is only about 18%, and the death rate rose by almost 3% per year.^2^ Up to now, the therapeutic strategies include surgical resection and orthotopic liver transplantation for early stage HCC, percutaneous ablation and chemoembolization for locoregional treatment and oral multikinase inhibitor sorafenib for advanced HCC.^3^ However, effective treatment for HCC is still limited owing to its high recurrence rates and frequent accompanying cirrhosis.^4^ It is urgent to develop innovative molecular therapies for better treatment of HCC.

MicroRNAs (miRNAs) are defined as small, evolutionarily conserved, non‐coding RNAs with 18‐25 nucleotides and able to regulate target genes by binding the 3ʹ‐untranslated region (3ʹ‐UTR).^5^, ^6^ They are widely involved in pathophysiological processes such as apoptosis, cell differentiation, cell proliferation and organ development,^7^ thus suppressing or promoting the progression of human cancers. MiR‐139a‐5p is functioned as a suppressor of HCC epithelial‐mesenchymal transition (EMT) and metastasis, and its down‐regulation is observed in HCC cells.^8^ Li et  al revealed the positive correlation between miR‐139 down‐regulation and poor HCC prognosis.^9^ Meanwhile, miR‐940 and miR‐193a‐5p have also been demonstrated to be remarkably down‐regulated in HCC tissues 10, 11. MiR‐940 suppresses tumour cell invasion and migration through regulating chemokine CXCR2,^12^ while miR‐193a decreases proliferation and increased apoptosis of HCC cells as well as improved the effect of sorafenib therapy.^13^ MiR‐193b increased cisplatin sensitivity of HCC cells and induced apoptosis.^14^ These previous studies suggest that miR‐139, miR‐940 and miR‐193 play negative roles in HCC development.

Cysteine‐rich acidic secreted protein/osteonectin, cwcv, and kazal‐like domains proteoglycan 1 (*SPOCK1*), the gene that encodes the protein core of a seminal plasma proteoglycan, is an oncogene that involves in cell‐cycle control, apoptosis, DNA repair and metastasis.^15^ According to Li et  al's study, *SPOCK1* is able to block apoptosis and promote metastasis in HCC.^16^ Its promotive effect was also found in various human malignancies, such as breast cancer,^17^ colorectal cancer,^18^ prostate cancer^18^ and ovarian cancer.^19^ Yan et  al investigated the relationship between miR‐129‐5p and *SPOCK1* and pointed out that *SPOCK1* can be regulated by miR‐129‐5p in gastric cancer, and the suppression of *SPOCK1* inhibits cancer deterioration.^20^ Therefore, overexpression of *SPOCK1* is adverse to cancer treatment. Since there are few researches at present to investigate the functions of *SPOCK1* in HCC, further studies about *SPOCK1* and its upstream regulators are essential.

In this study, the expression levels of miR‐139‐5p, miR‐940 and miR‐193a‐5p in HCC were investigated and their biological functions were explored. The target relationships between these miRNAs and *SPOCK1* were also investigated to uncover the mechanisms that underlie miRNAs' influence on HCC development. The results could provide novel insights into potential molecular targets for HCC treatment.

## 2 MATERIALS AND METHODS

### 2.1 Patient samples

This study was approved by the Human Research Ethics Committee of the First Affiliated Hospital of Guangzhou University of Chinese Medicine. Moreover, the experiments were undertaken with the understanding and written consent of each subject. Forty‐six pairs of HCC and matched noncancerous liver tissues were obtained from the First Affiliated Hospital of Guangzhou University of Chinese Medicine. The tissues were from untreated patients undergoing surgery and diagnosed by pathologists before being preserved at −80°C. The pathological characteristic parameters of the patients were shown in Table 1.

**Table 1:** Clinical and pathologic characteristics of 46 patients with HCC

Characteristics	miR‐139‐5p high	miR‐139‐5p low	*P* value	miR‐139‐5p high	miR‐139‐5p low	*P* value	miR‐139‐5p high	miR‐139‐5p low	*P* value
	n = 10	n = 36		n = 9	n = 37		n = 7	n = 39	
Gender									
Male	4	21	0.3032	4	21	0.506	5	19	0.268
Female	6	15		5	16		2	20	
Age									
＜55	6	19	0.685	6	20	0.4936	4	25	0.7254
≥55	4	17		3	17		3	14	
Tumour grade									
G1	5	10	0.2079	5	10	0.2066	3	9	0.2289
G2	5	19		2	19		4	19	
G3	0	7		2	8		0	11	
AFP									
≤20 μg/L	7	6	**0.0009** **	6	17	0.2648	5	16	0.2
＞20 μg/L	3	30		3	20		1	13	
Lymph node metastasis									
Absence	6	20	0.802	6	22	0.6911	7	27	0.0878
Presence	4	16		3	15		0	12	
Distant metastasis									
Absence	10	25	0.0681	9	28	0.099	7	35	0.3752
Presence	0	9		0	9		0	4	

The bold type indicates statistical significance.

Data were compared by Chi‐square test.

AFP: alpha‐fetoprotein; HCC: hepatocellular carcinoma.

** *P *< 0.01.

### 2.2 Microarray analysis

The transcriptome profiles of 16 liver specimens, including nine specimens from the tumour area (HCC) and seven specimens from normal liver (NL) of patients who underwent hepatic resection, were determined using the Affymetrix Human miRNA Array 2.0 (HTA 2.0) GeneChips (Affymetrix) and analysed by GPL570 platform. A volcano plot filtering (fold change > 2, *P* < 0.05) was performed between HCC and NL specimens to identify the differentially expressed miRNAs in HCC tissues. Hierarchical clustering was performed based on the differential expressions of miRNAs. The target genes of miRNAs were screened out by TargetscanHuman 7.1 (http://www.targetscan.org/vert_71/), starBase v2.0 (http://starbase.sysu.edu.cn/), and microRNA.org (http://www.microrna.org/). The common target gene of these miRNAs was selected by Venn diagram.

### 2.3 Kaplan‐Meier survival analysis

The mRNA/miRNA normalized gene expression data and clinical information of 377 liver hepatocellular carcinoma (LIHC) samples were obtained from the Cancer Genome Atlas (TCGA) database (https://portal.gdc.cancer.gov/projects/TCGA-LIHC). These patients were divided into two groups according to the median value of expression level of three selected miRNAs and *SPOCK1*. All the data were applied to perform Kaplan‐Meier survival analysis and log‐rank statistics. Hazard ratio (HR) and 95% CI of the association between *SPOCK1*/miRNAs and overall survival were calculated using Cox proportional hazards analysis with R “Survival”, “Survplot”, and “Kmplot” packages.

### 2.4 Cell line and transfection

The HCC cell line HepG2, Hep3b and NL cell line HL‐7702 were purchased from BeNa Culture Collection (Beijing, China). HepG2 and Hep3b cells were grown in high‐glucose DMEM (Invitrogen, Carlsbad, CA, USA) supplemented with 10% foetal bovine serum (FBS, Invitrogen), 100 U/mL penicillin and 100 µg/mL streptomycin. HL‐7702 cells were immortalized cell line and maintained in RPMI‐1640 (Invitrogen) containing 10% FBS. All the cells were cultured at 37°C with 5% CO_2_.

Transfections of HepG2 and Hep3b cells were performed by using Lipofectamine™ 3000 (Invitrogen). HepG2 and Hep3b cells transfected with miRNA mimics were divided into four groups: negative control (NC), miR‐139‐5p, miR‐940, miR‐193a‐5p and cotransfection groups that being transfected with miR‐NC, miR‐139‐5p mimics, miR‐940 mimics, miR‐193a‐5p mimics and three miRNA mimics respectively. MiRNA mimics and miR‐NC were all obtained from Sangon Biotech (Shanghai, China). *SPOCK1* overexpression was constructed by inserting full‐length *SPOCK1* (generated from HepG2 cDNA) into the pcDNA3.1 vector (Life Technologies, Gaithersburg, MD, USA). Si‐*SPOCK1* was synthesized by GenePharma (Shanghai, China). HepG2 cells with *SPOCK1* overexpression/si‐*SPOCK1* were divided into seven groups: Blank group with untreated HepG2 cells; NC group transfected with irrelevant sequence; *SPOCK1* group transfected with pcDNA3.1‐*SPOCK1*; si‐SPOCK1 group transfected with si‐*SPOCK1*; *SPOCK1 *+ miR‐139‐5p, *SPOCK1 *+ miR‐940 and *SPOCK1 *+ miR‐193‐5p groups transfected with pcDNA3.1‐*SPOCK1* and miR‐139‐5p mimics, miR‐940 mimics and miR‐193‐5p mimics respectively.

### 2.5 qRT‐PCR

Total RNA isolated by TRIzol reagent (Life Technologies) was quantified by NanoDrop ND‐1000 Sepctrophotometer (Thermo Fisher Scientific, Waltham, MA, USA). SuperScript III First‐Strand Synthesis System kit (Invitrogen) and SoFast EvaGreen Supermix (Bio‐Rad, Hercules, CA) were applied to reversely transcript mRNA into cDNA, while NCode™ VILO™ miRNA cDNA Synthesis kit (Life Technologies) was used for miRNA reverse transcription. qPCR of mRNA and miRNA was performed by SoFast™ EvaGreenH Supermix (Bio‐Rad) and EXPRESS SYBR Green ER miRNA quantitative real time polymerase chain reaction (qRT‐PCR) kit (Life Technologies) respectively. Primers used are listed in Table 2. Reduced glyceraldehyde‐phosphate dehydrogenase (GAPDH) and U6 were internal controls. The relative expression was expressed by $2^{-\Delta \Delta C_{t}}$.

**Table 2:** Primer Sequences for qRT‐PCR

Genes		Sequence (5ʹ‐3ʹ)
miR‐139‐5p	Sense	TCTACAGTGCACGTGTC
	Antisense	GAATACCTCGGACCCTGC
miR‐940	Sense	AAGGCAGGGCCCCC
	Antisense	GAATACCTCGGACCCTGC
miR‐193a‐5p	Sense	TGGGTCTTTGCGGGC
	Antisense	GAATACCTCGGACCCTGC
*SPOCK1*	Sense	CAGCCTGTCCACACAAAAGC
	Antisense	CCATCGATTTGGGGGTTCCA
GAPDH	Sense	GTCAACGGATTTGGTCTGTATT
	Antisense	CGCUUCACGAAUUUGCGUGUCAU
U6	Sense	CTCGCTTCGGCAGCACATA
	Antisense	AACGATTCACGAATTTGCGT

### 2.6 Dual luciferase reporter assay

*SPOCK1* 3ʹ‐UTR and the mutated control were cloned into the plasmid vector pmirGLO. MiRNA mimics (miR‐139‐5p mimics, miR‐940 mimics and miR‐193a‐5p mimics) were then transfected into HepG2 cells containing wild‐ or mutant‐type 3ʹ UTR pmirGLO plasmids by using Lipofectamine^TM^ 3000 (Invitrogen). Dual‐Luciferase Assay System from Promega (Madison, WI, USA) was used to measure the activities of firefly luciferase and Renilla luciferase in the cell lysates. PmirGLO, miRNA mimics and NC were all obtained from Promega.

### 2.7 RNA pull‐down assay

RNA structure buffer (100 μL) was used to incubate biotin‐labelled RNA (3 μg), that is, Bio‐NC‐probe, Bio‐*SPOCK1*‐probe, Bio‐*GDE1*‐probe, Bio‐*TRAF3*‐probe and Bio‐*TAOK1*‐probe. The incubation conditions were 90°C for 5 minutes, ice bath for 5 minutes and in room temperature for 20 minutes. Approximately 10^7 ^HepG2 cells were collected and added with 1 mL TRIzol reagent (Life Technologies) to extract total RNA. Then, 1 mg total RNA was mixed with biotin‐labelled RNA, and the mixture was incubated with prewashed streptavidin beads for 1 hour before being centrifuged (1006.2 *g*, 4°C, 3 minutes) to separate beads. The beads were washed to remove non‐specific binding. The hybridized RNA remained was reversely transcripted and quantified by SuperScript III First‐Strand Synthesis System kit (Invitrogen) and Maxima SYBR Green qPCR Master Mix (2X) kit (Thermo Scientific) with GAPDH as internal control.

### 2.8 MTT assay

HepG2 cells transfected for 1, 2, 3 and 4 days were seeded into 96‐well plates, with 20‐μL 3‐(4,5‐dimethyl‐2‐thiazolyl)‐2,5‐diphenyl‐2‐H‐tetrazolium bromide,thiazolyl blue tetrazolium bromide (MTT) (Servicebio, Wuhan, China) in each well for further incubation. After 4 hours, the liquid in each well was replaced with 100 μL dimethyl sulphoxide (DMSO). The samples were shaken slightly for 10 minutes. The microplate reader was used to measure absorbance at 490 nm.

### 2.9 Transwell assay

Matrigel (BD Biosciences, San Jose, CA, USA) diluted with serum‐free medium was placed into transwell chamber (50 μL/well) for analysing cell invasion. The upper chamber was seeded with HCC cells (1 × 10^6^ cells/well), while the lower one was added with 500 μL DMEM (supplemented with 10% FBS). After 24 hours incubation, the medium and cells remaining in upper chamber were removed. Fixation and staining of invaded cells in lower chamber were performed by 4% paraformaldehyde (20 minutes) and 0.1% crystal violet (30 minutes). The number of invaded cells was counted by microscope.

### 2.10 Cell apoptosis

Cells harvested after 48 hours transfection were stained by PI Annexin V Apoptosis Detection kit (BD Bioscience) and then detected by FACS Calibur flow cytometry (BD Bioscience) to determine apoptosis. Data analysis was performed by FACS Diva software.

### 2.11 Tumour formation in vivo

Six 8‐week‐old male BALB/c‐nude mice per group were used for this experiment. HepG2 cell suspension was prepared using logarithmic phase cells. The miR‐139‐5p, miR‐940 and miR‐193a‐5p mature sequences were first cloned after the cytomegalovirus promoter into the pcDNA3.1 vector. Then, the vectors were transfected into HepG2 cells as mentioned previously for G418 (Geneticin) selection. The suspension was subcutaneously injected into the back of nude mice (2 × 10^6 ^cells per mouse) after selection. The mice were killed after 4 weeks and the tumour weights were measured. The tumour volumes were calculated every week by the formula S = 1/2 × *ab* ^2^ (*a* is tumour length and *b* is tumour width). All animal experiments were approved by the First Affiliated Hospital of Guangzhou University of Chinese Medicine.

### 2.12 Western blot

Tumour tissues obtained from killed nude mice were grinded into powder in liquid nitrogen with RIPA buffer (Solarbio, Beijing, China). Total proteins in tissue were extracted by protein extraction kit (Millipore, Billerica, MA, USA) separated by electrophoresis on 10% SDS‐PAGE. After transferring the protein onto polyvinylidene fluoride membrane (Invitrogen), the membrane was in turn incubated with primary antibody overnight at 4°C and secondary antibody for 1 hour. The primary antibodies were rabbit anti‐human antibodies: anti‐SPOCK1 (1:2000, ab229935), anti‐Ki67 (1:1000, ab92742), anti‐caspase 3 (1:500, ab13847), anti‐caspase 7 (1 µg/mL, ab69540), anti‐caspase 8 (1 µg/mL, ab25901), anti‐E Cadherin (1:500, ab15148), anti‐E Cadherin (1:500, ab15148), anti‐N Cadherin (1 µg/mL, ab18203), anti‐Vimentin (1 µg/mL, ab45939) and anti‐GAPDH (1:2500, ab9485) and mouse anti‐human antibodies: anti‐PCNA (1 µg/mL, ab29) and anti‐GAPDH (1 µg/mL, ab9484) and the secondary antibody was goat anti‐rabbit IgG‐HRP (1:10000, ab6721) and goat anti‐mouse IgG‐HRP (1:5000, ab6789). The antibodies used were purchased from Abcam (Cambridge, MA, USA). Electrochemiluminescent (ECL) Detection System (Thermo Scientific) was used for signal detection.

### 2.13 Statistics

All statistical analyses were performed using GraphPad Prism 6.0. Data were expressed as mean ± SD. Student's *t* test and one‐way ANOVA were used to compare the significance between two groups and among multiple groups respectively. Two‐sided *P* values were calculated, and *P* < 0.05 was chosen for statistical significance. All experiments were repeated for at least three times.

## 3 RESULTS

### 3.1 MiR‐139‐5p, miR‐940 and miR‐193a‐5p were down‐regulated in HCC tissues and HepG2 cell

**Figure 1:**
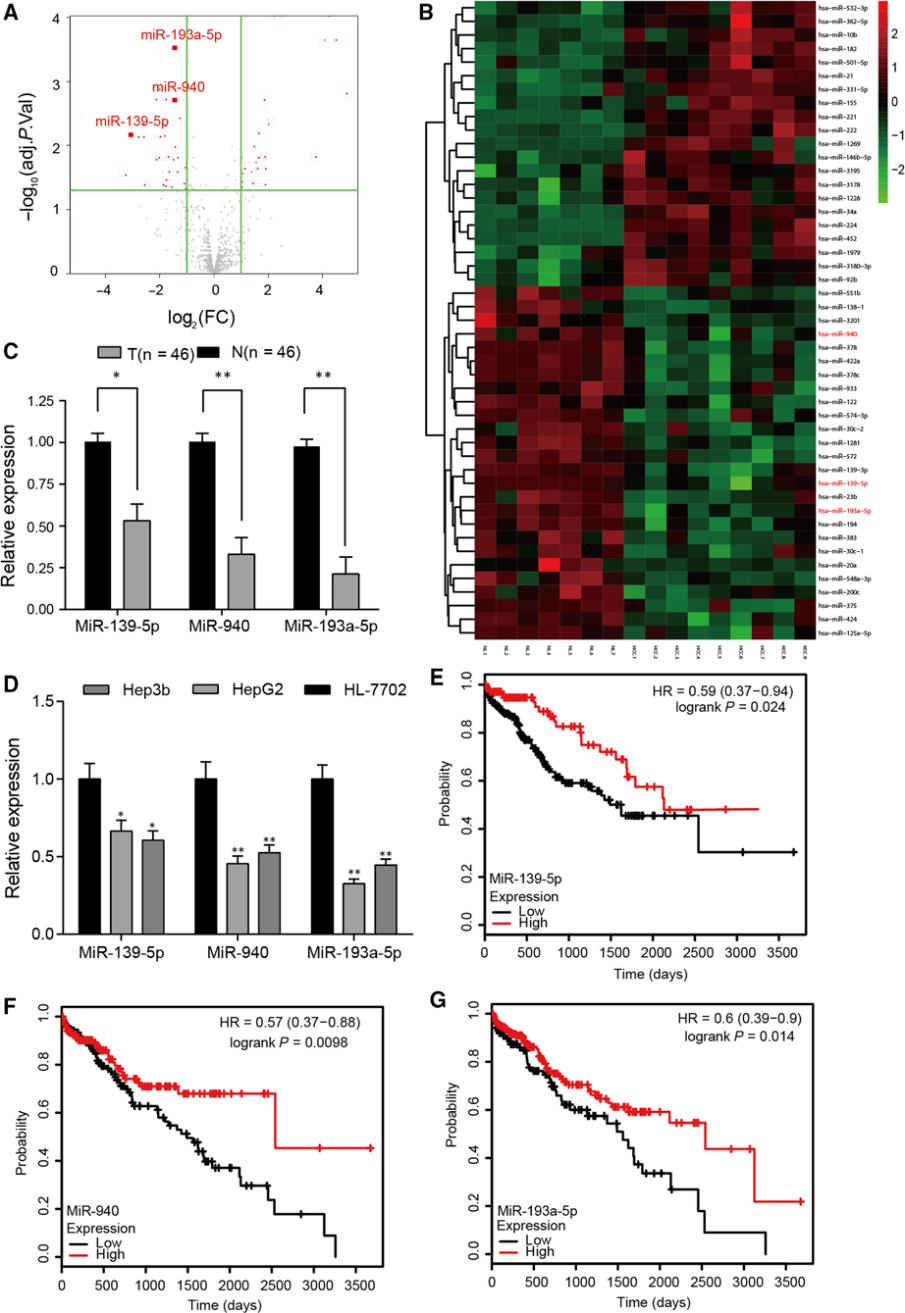
MiR‐139‐5p, miR‐940 and miR‐193a‐5p were down‐regulated in hepatocellular carcinoma (HCC) tissues and HepG2 cell. A, Volcano plot reflected differentially expressed miRNAs with fold change >2 and *P* < 0.05 in HCC tissues, including miR‐139‐5p, miR‐940 and miR‐193a‐5p. B, Heat map reflected the low expressions of miR‐139‐5p, miR‐940 and miR‐193a‐5p in HCC tissues. NL was NL tissue and HCC was HCC tissue. (C) Relative expressions of miR‐139‐5p, miR‐940 and miR‐193a‐5p in 46 HCC tissues detected by qRT‐PCR were significantly lower than those in 46 matched adjacent tissues. N was adjacent tissue and T was HCC tissue. D, Relative expressions of miR‐139‐5p, miR‐940 and miR‐193a‐5p in HepG2 and Hep3b cells detected by qRT‐PCR were significantly lower than those in HL‐7702 cell. E, Kaplan‐Meier curve analysis of overall patient survival between those with high miR‐139‐5p expression and low miR‐139‐5p expression in the TCGA‐LIHC database. F, Kaplan‐Meier curve analysis of overall patient survival between those with high miR‐940 expression and low miR‐940 expression in the TCGA‐LIHC database. G, Kaplan‐Meier curve analysis of overall patient survival between those with high miR‐193a‐5p expression and low miR‐193a‐5p expression in the TCGA‐LIHC database. N was adjacent tissue and T was HCC tissue. *P < 0.05, **P < 0.01 compared with adjacent tissues or normal HL‐7702 cell line

MiRNAs with differential expression (fold change > 2, *P* < 0.05) in HCC tissues were screened out (Figure 1A). MiR‐139‐5p, miR‐940 and miR‐193a‐5p were found to target multiple common genes in human cancers, which could affect cancer development, thus were chosen as the miRNAs of interest in this study. Compared with NL tissues, their expression levels were aberrantly lower in HCC tissues (Figure 1B). The relative expressions in 46 pairs of HCC and matched adjacent tissues confirmed the down‐regulation of miR‐139‐5p, miR‐940 and miR‐193a‐5p in tumour tissues (Figure 1C). According to Table 1, the expressions of miR‐139‐5p, miR‐940 and miR‐193a‐5p were low in most HCC patients, but the correlation between these miRNAs and pathological characteristic parameters such as tumour grade, alpha‐fetoprotein (AFP) level and metastasis were not obvious, which might because of the small size of the samples. The expression of miR‐139‐5p, miR‐940 and miR‐193‐5p was lower in HepG2 and Hep3b cell lines compared with normal HL‐7702 cell lines (Figure 1D). Kaplan‐Meier survival analysis based on TCGA database indicated that higher expression levels of miR‐139‐5p/miR‐940/miR‐193a‐5p significantly correlated with longer survival of HCC patients (Figure 1E‐G).

### 3.2 MiR‐139‐5p, miR‐940 and miR‐193a‐5p restrained growth and invasion of HCC cells

**Figure 2:**
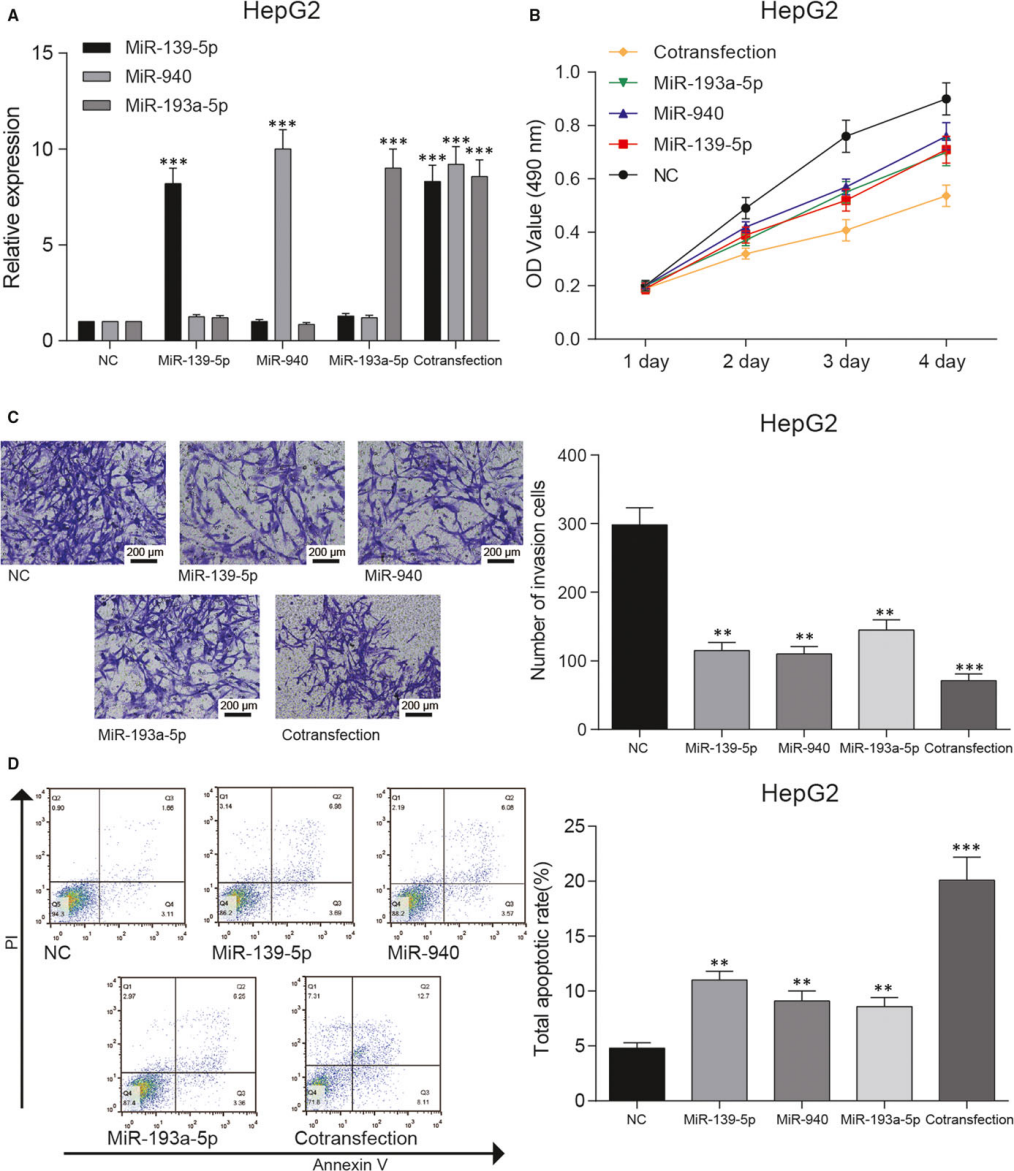
MiR‐139‐5p, miR‐940 and miR‐193a‐5p restrained growth and invasion of HepG2 cells. A, Relative expressions of miR‐139‐5p, miR‐940 and miR‐193a‐5p were elevated in hepatocellular carcinoma cell line HepG2 after being transfected with miR‐139‐5p mimics, miR‐940 mimics and miR‐193a‐5p mimics respectively. (B,C) Cell viability of HepG2 cells detected by MTT assay and number of invasion cells detected by transwell assay decreased in miR‐139‐5p, miR‐940 and miR‐193a‐5p groups. D, Total apoptotic rate of HepG2 cells detected by flow cytometry increased in miR‐139‐5p, miR‐940 and miR‐193a‐5p groups. **P < 0.01, ***P < 0.01 compared with NC group

**Figure 3:**
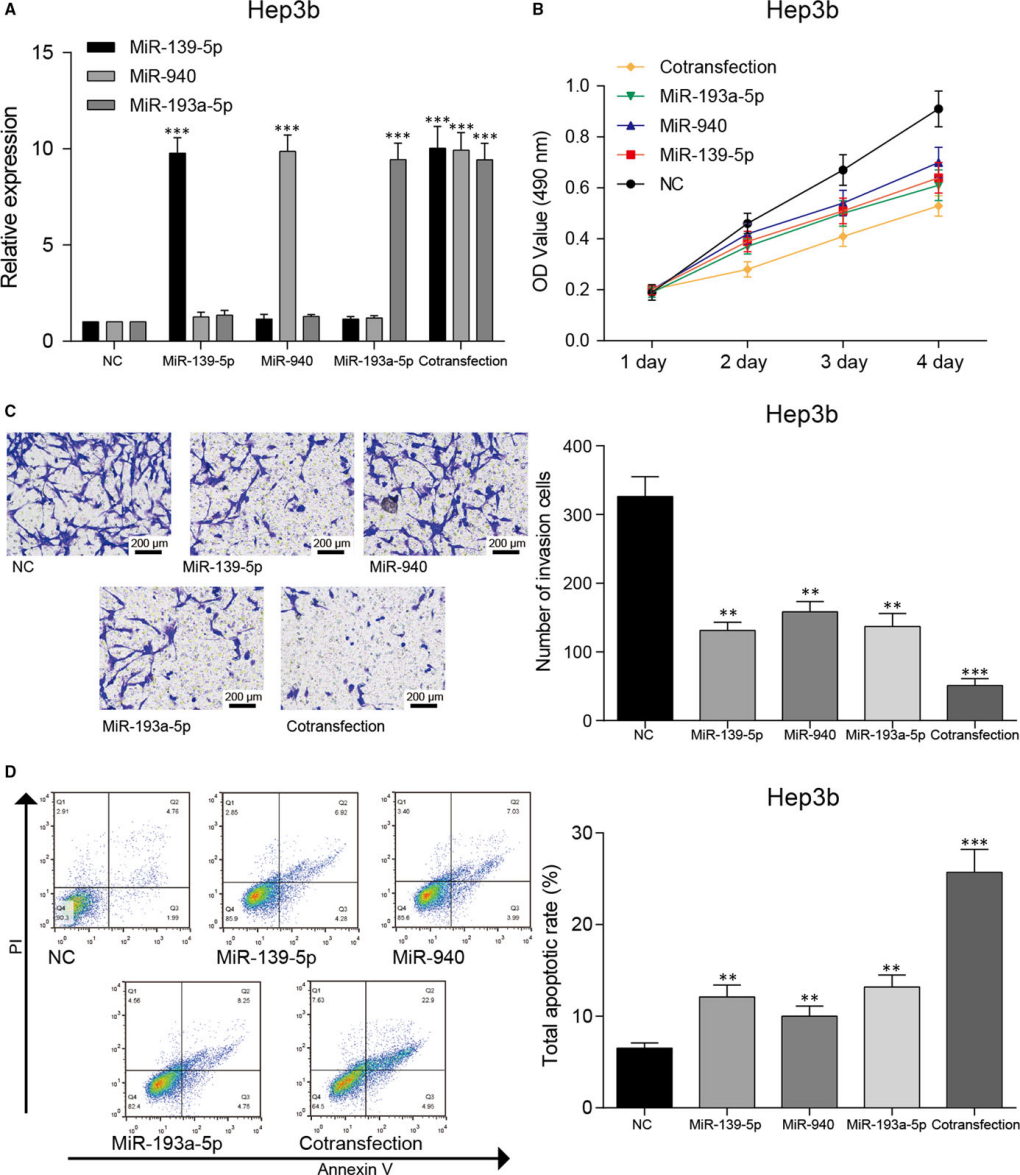
MiR‐139‐5p, miR‐940 and miR‐193a‐5p restrained growth and invasion of Hep3b cells. A, qRT‐PCR was used to evaluate the expression of miRNAs after transfection. B, Cell viability was detected by MTT assay. C, The ability of invasion was detected by transwell assay. D, Total apoptotic rate of Hep3b cells accessed by flow cytometry assay. **P < 0.01, ***P < 0.01 compared with NC group

Transfection of miRNA mimics up‐regulated the expression of miR‐139‐5p, miR‐940 and miR‐193a‐5p in HCC cell line HepG2 and Hep3b. In addition, cotransfection of miR‐139‐5p, miR‐940 and miR‐193a‐5p mimics enhanced the expression of these miRNAs, which indicated that there was no interference among them (Figures 2A & 3A). Overexpression of these miRNAs significantly reduced viability and invasion of HepG2 and Hep3b cells, while cotransfection of three miRNAs attenuated viability and invasion of HCC cells more significantly (Figures 2B,C & 3B,C). In contrast, apoptosis of HepG2 and Hep3b cells was induced after up‐regulating the expression of miR‐139‐5p, miR‐940 and miR‐193a‐5p. Furthermore, contransfection of miR‐139‐5p, miR‐940 and miR‐193a‐5p showed more obvious pro‐apoptotic effect on HCC cells (Figures 2D & 3D). These results indicated that miR‐139‐5p, miR‐940 and miR‐193a‐5p negatively affected HCC cell growth and invasion.

### 3.3 *SPOCK1 *was the common target gene of miR‐139‐5p, miR‐940 and miR‐193a‐5p

**Figure 4:**
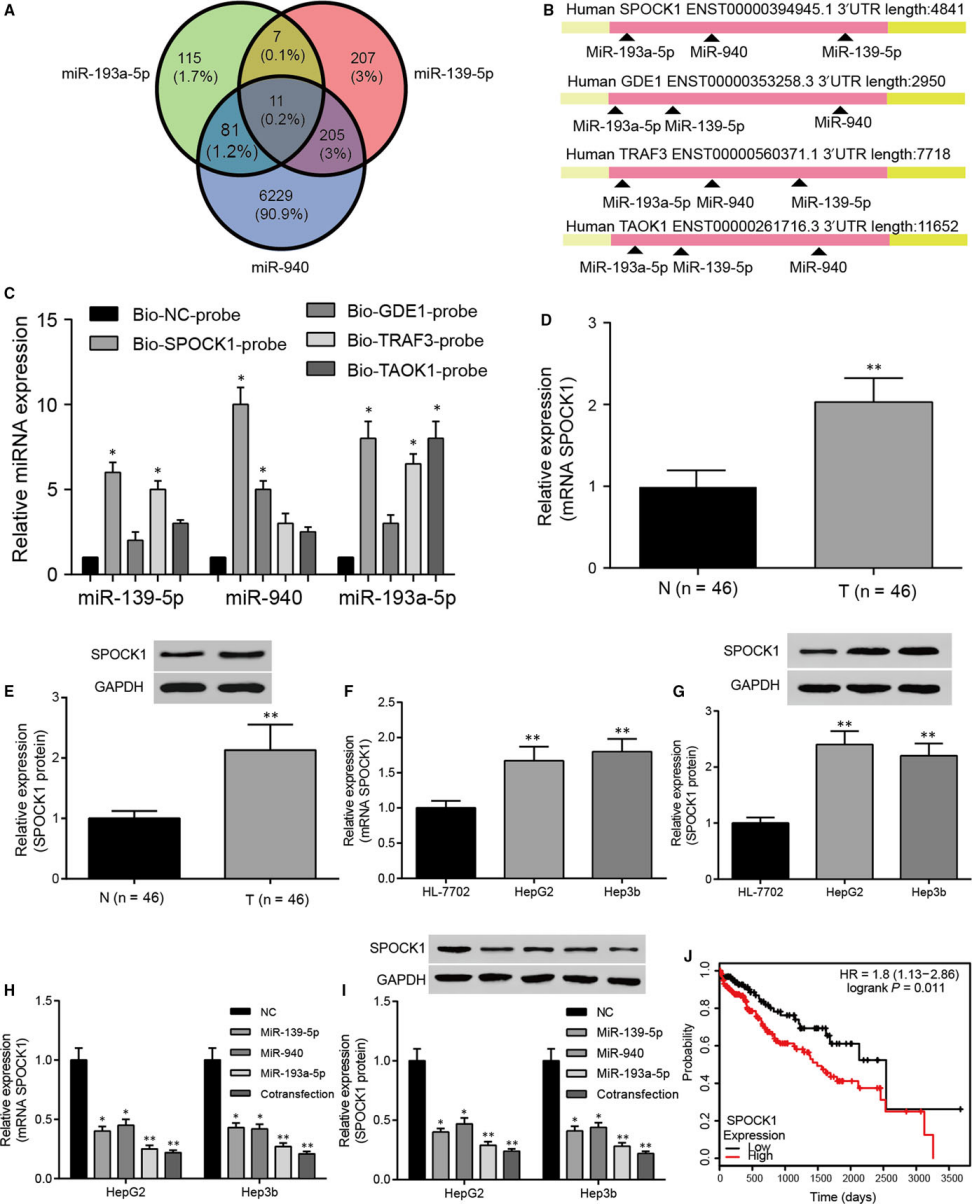
SPOCK1 was up‐regulated in hepatocellular carcinoma (HCC) tissues and HepG2 cell. A, Eleven common target genes of miR‐139‐5p, miR‐940 and miR‐193a‐5p were selected by Venn diagram. (B) *SPOCK1, GDE1*, *TRAF3* and *TAOK1* were common target genes of miR‐139‐5p, miR‐940 and miR‐193a‐5p. C, According to RNA pull‐down assay, the three selected miRNAs expressions of Bio‐*SPOCK1* probes were all increased. *P < 0.05 compared with Bio‐NC‐probe. D, The relative mRNA expression of *SPOCK1* detected by qRT‐PCR was higher in HCC tissues. **P < 0.01 compared with normal tissues. E, The relative protein expression of SPOCK1 detected by western blot was higher in HCC tissues. **P < 0.01 compared with normal tissues. F, The relative mRNA expression of *SPOCK1* detected by qRT‐PCR was higher in HepG2 and Hep3b cell lines. **P < 0.01 compared with normal HL‐1107 cell line. G, The relative protein expression of SPOCK1 detected by western blot was higher in HepG2 and Hep3b cell lines. **P < 0.01 compared with normal HL‐1107 cell line. H, The relative mRNA expression of *SPOCK1* detected by qRT‐PCR was reduced after overexpression of miR‐139‐5p/miR‐940/miR‐193a‐5p.*P < 0.05, **P < 0.01 compared with NC group. I, The relative protein expression of SPOCK1 detected by western blot was reduced after overexpression of miR‐139‐5p/miR‐940/miR‐193a‐5p. *P < 0.05, **P < 0.01 compared with NC group. J, Kaplan‐Meier curve analysis of overall patient survival between those with high *SPOCK1* expression and low *SPOCK1* expression in the TCGA‐LIHC database.

The possible target genes of miR‐139‐5p, miR‐940 and miR‐193‐5p were predicted by Targetscan database. Among them, 11 common target genes were screened out through the analysis of the potential target genes profile (Figure 4A). Then, we used another two databases, starBase v2.0 and microRNA.org, to verify our prediction. By literature review, we have confirmed four predicted common target genes, *SPOCK1*, *GDE1*, *TRAF3* and *TAOK1*, that were frequently found associated with tumourigenesis. We then conducted RNA pull‐down assay to determine the binding relationship between the three miRNAs and the four genes. Although all of them were predicted to be able to bind to miR‐139‐5, miR‐940 and miR‐193a‐5p (Figure 4B), only *SPOCK1* had strong binding capacity with all three selected miRNAs (Figure 4C). In 46 pairs HCC tissues, SPOCK1 mRNA and protein expressions were also distinctively elevated (Figure 4D,E). In addition, the mRNA and protein expression levels of SPOCK1 were also higher in HepG2 and Hep3b cells (Figure 4F,G). After miR‐139‐5p/miR‐940/miR‐193a‐5p was overexpressed, the level of SPOCK1 was restrained than NC group both in mRNA and protein expression. However, in cotransfection group, the expression of SPOCK1 mRNA and protein was lower than those in single miRNA mimics group, but the difference was not significant (Figure 4H,I). The *SPOCK1* high expression group had a significantly poorer overall survival by Kaplan‐Meier analysis (Figure 4J). We supposed that these miRNAs have other target mRNAs which are tumour promoter and overexpression of these miRNAs inhibits these mRNAs the expression of these mRNAs leading to a better antitumour effect. Because of the limited expression of SPOCK1, cotransfection of three miRNAs does not further attenuate the level of SPOCK1.

**Figure 5:**
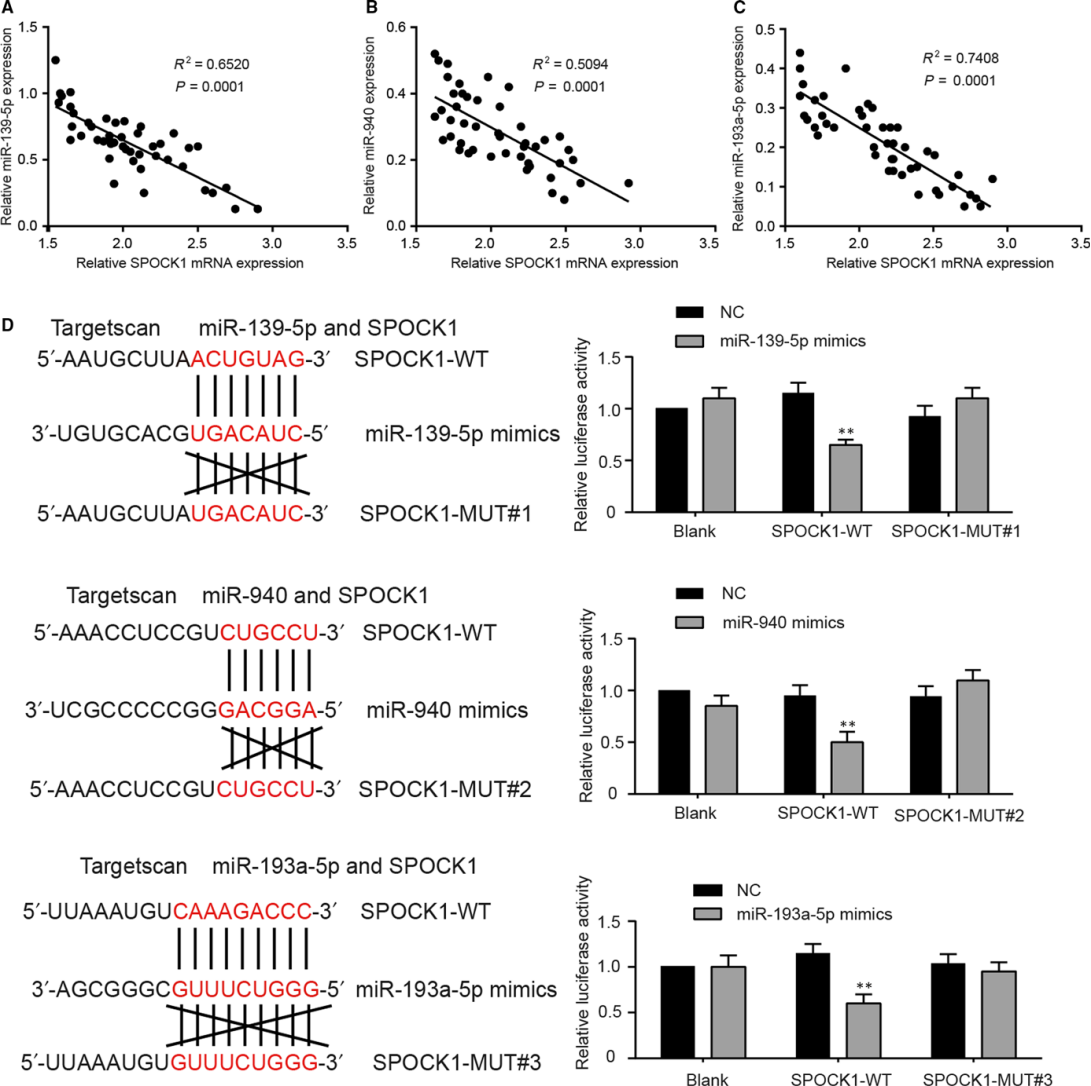
*SPOCK1 *was the common target gene of miR‐139‐5p, miR‐940 and miR‐193a‐5p. A, Correlation analysis indicated that the expression of miR‐139‐5p was negatively correlated with *SPOCK1* level. B, Correlation analysis indicated that the expression of miR‐940 was negatively correlated with *SPOCK1* level. C, Correlation analysis indicated that the expression of miR‐193a‐5p was negatively correlated with *SPOCK1* level. D, According to dual luciferase reporter assay, the relative luciferase activities of HepG2 cells in *SPOCK1*‐WT + miRNA mimics (miR‐139‐5p mimics, miR‐940 mimics and miR‐193a‐5p mimics) groups were significantly decreased, while those in *SPOCK1*‐MUT + miRNA mimics groups showed no significant variation. **P < 0.01 compared with NC + SPOCK1‐WT group.

The results of the correlation studies among the three miRNAs and *SPOCK1* expression form the 46 pairs of HCC and matched noncancerous liver tissues which we collected indicated that miR‐139‐5p/miR‐940/miR‐193a‐5p expressions were negatively correlated with *SPOCK1* expression (Figure 5A‐C). The target relationship between *SPOCK1* and three miRNAs was further confirmed by the decreased luciferase activity of HepG2 cells co‐transfected with wild‐type *SPOCK1*‐3ʹ UTR and miRNA mimics. Moreover, miR‐193a‐5p had more binding sites with *SPOCK1* compared with other two miRNAs (Figure 5D). In brief, *SPOCK1* could closely bind to miR‐139‐5p, miR‐940 and miR‐193‐5p as their common target gene.

### 3.4 *SPOCK1 *promoted growth and invasion of HCC cells

**Figure 6:**
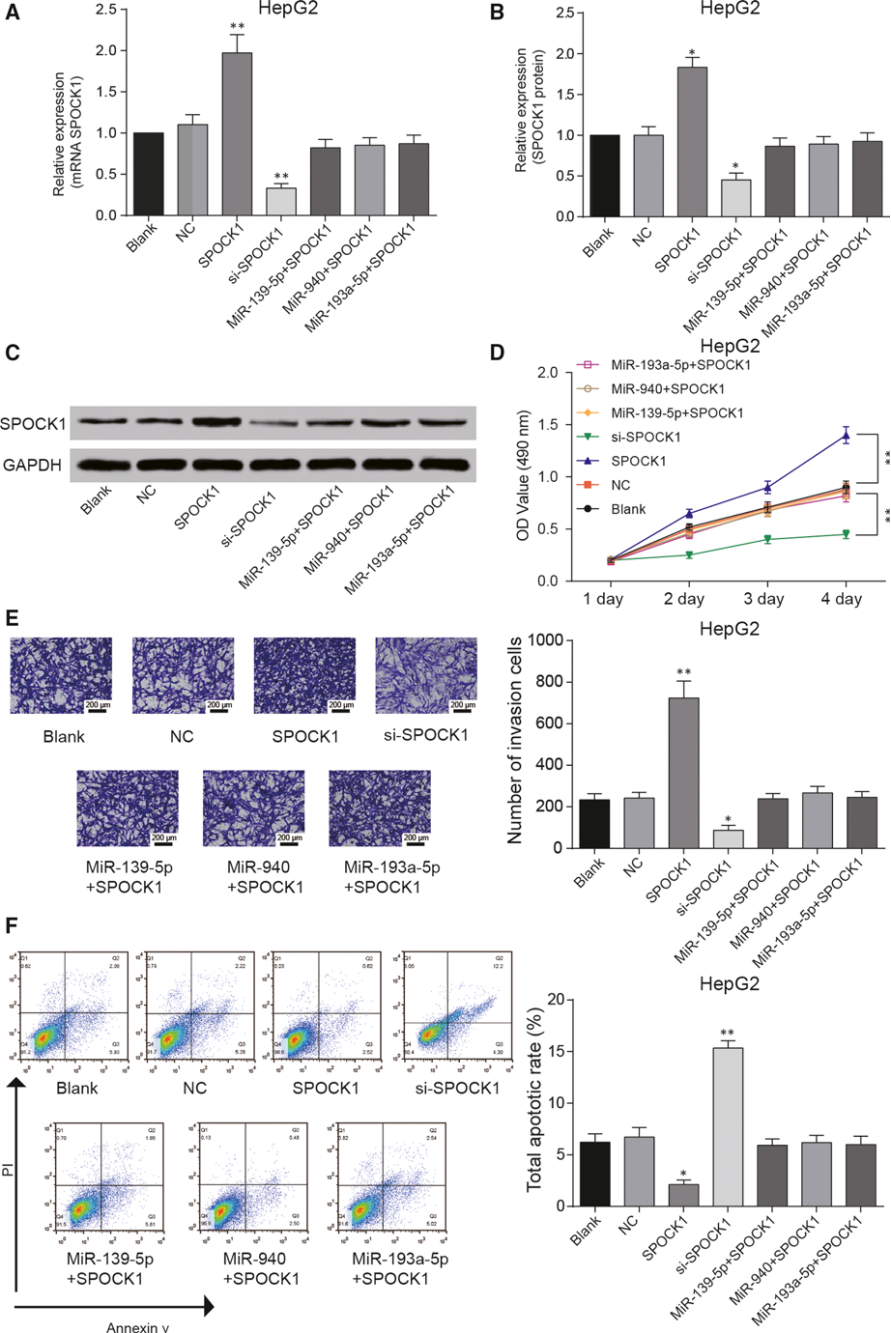
*SPOCK1 *promoted growth and invasion of HepG2 cells. A, The relative expression of *SPOCK1* in HCC cells detected by qRT‐PCR increased in *SPOCK1* group, reduced in si‐*SPOCK1* group and remained similar as that in NC group in *SPOCK1 *+ miRNAs (miR‐139‐5p, miR‐940 and miR‐193a‐5p) groups. (B,C) Western blot was used to evaluate the expression of SPOCK1 protein. (D,E) Cell viability of HepG2 cells detected by MTT assay and number of invasion cells detected by transwell assay increased in *SPOCK1* group, reduced in si‐*SPOCK1* group and almost remained unchanged in *SPOCK1 *+ miRNAs (miR‐139‐5p, miR‐940 and miR‐193a‐5p) groups. F, Total apoptotic rate of HepG2 cells detected by flow cytometry decreased in *SPOCK1* group, increased in si‐*SPOCK1* group, whereas had no significant change in *SPOCK1 *+ miRNAs (miR‐139‐5p, miR‐940 and miR‐193a‐5p) groups. *P < 0.05, **P < 0.01 compared with NC group

**Figure 7:**
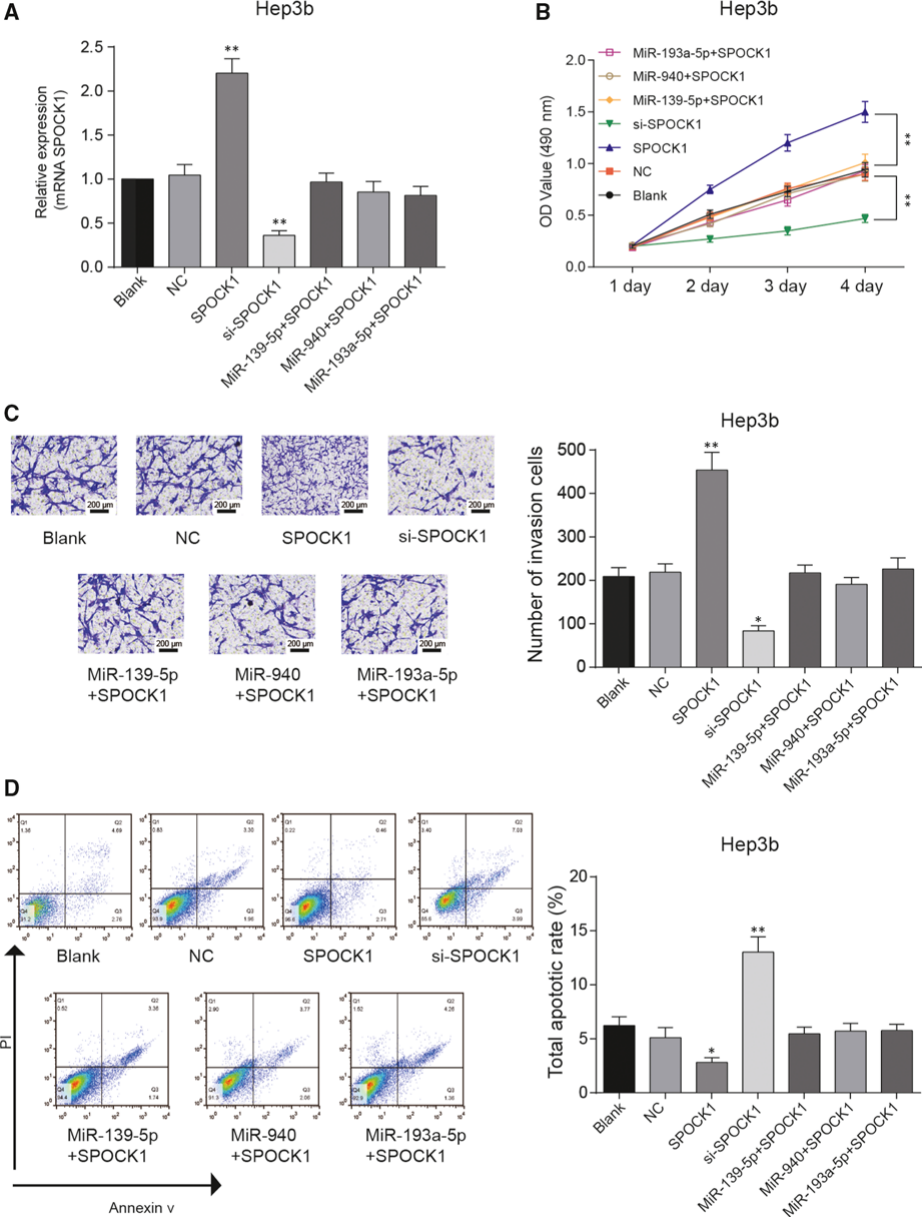
*SPOCK1 *promoted growth and invasion of Hep3b cells. A, qRT‐PCR was used to detect the expression of *SPOCK1*. B, Cell viability of Hep3b cells was detected by MTT assay. C, Transwell assay revealed the invasion ability of Hep3b cells. D, Flow cytometry was performed to access the apoptosis rate of Hep3b cells

Overexpression of *SPOCK1* in HCC cells was achieved by transfection, si‐*SPOCK1* inhibited the expression of *SPOCK1* and the high expression of *SPOCK1* could be rescued by miR‐139‐5p, miR‐940 and miR‐193a‐5p (Figures 6A & 7A); the protein expression of SPOCK1 was similar with tendency of *SPOCK1* mRNA expression (Figure 6B,C). *SPOCK1* overexpression promoted the proliferation and invasion of HepG2 and Hep3b cells, while si‐*SPOCK1* inhibited the proliferation and invasion of HepG2 and Hep3b cells. However, simultaneous overexpression of *SPOCK1* and miRNAs retrieved the viability and invasive ability of HCC cells (Figures 6D,E & 7B,C). Meanwhile, cell apoptosis was also suppressed by *SPOCK1* overexpression and remained stable in *SPOCK1*+miRNAs groups, si‐*SPOCK1* promoted the cell apoptosis (Figures 6F & 7D). In short, *SPOCK1* could promote growth and invasion of HCC cells, and its promotive effect could be attenuated by miR‐139‐5p, miR‐940 and miR‐193a‐5p.

### 3.5 MiR‐139‐5p, miR‐940 AND miR‐193a‐5p inhibited tumour growth in vivo

**Figure 8:**
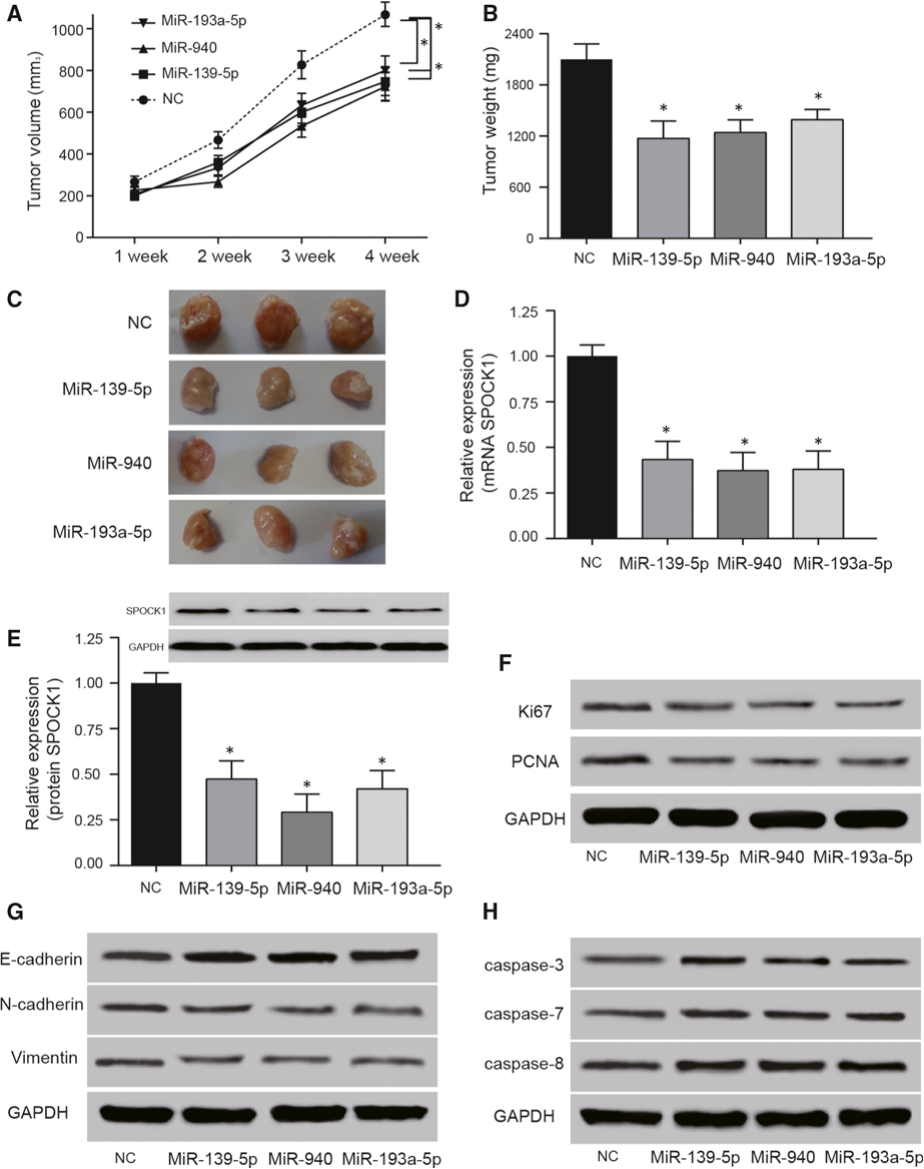
MiR‐139‐5p, miR‐940 and miR‐193a‐5p inhibited tumour growth in vivo. (A,B) The volume and weight of liver tumours of nude mice were significantly decreased in miR‐139‐5p, miR‐940 and miR‐193a‐5p groups. C, The tumours were taken out from killed nude mice after 4 wk. Tumours in NC group were obviously larger than those in miRNAs groups. (D,E) Relative *SPOCK1* mRNA expression detected by qRT‐PCR and protein expression detected by western blot were decreased in miR‐139‐5p, miR‐940 and miR‐193a‐5p groups. *P < 0.05, compared with NC group. F, Relative proliferation markers including PCNA and Ki67 expression detected by western blot were reduced in miR‐139‐5p, miR‐940 and miR‐193a‐5p groups. G, The relative EMT marker E‐cadherin detected by western blot was increased and N‐cadherin and Vimentin expressions were reduced in miR‐139‐5p, miR‐940 and miR‐193a‐5p group. H, Relative cell apoptosis regulators including caspase 3, caspase 7 and caspase 8 detected by western blot were increased in miR‐139‐5p, miR‐940 and miR‐193a‐5p group

Transfection of miR‐139‐5p mimics, miR‐940 mimics and miR‐193a‐5p mimics in HepG2 cells reduced the volume and weight of liver tumours in vivo (Figure 8A‐C). The *SPOCK1* expression in these tumour tissues showed that overexpression of miRNAs significantly decreased the expression of *SPOCK1* (Figure 8D). The protein expression of SPOCK1 in tumour tissues confirmed the down‐regulation of *SPOCK1* resulted from miR‐139‐5p, miR‐940 and miR‐193a‐5p (Figure 8E). Consistently, miR‐139‐5p/miR‐940/miR‐193a‐5p mimics reduced the PCNA and Ki67 expression which were highly expressed in proliferating cells (Figure 8F). The ectopic overexpression of miR‐139‐5p/miR‐940/miR‐193a‐5p promoted the protein levels of E‐cadherin, while reducing N‐cadherin and Vimentin in the tumour tissues (Figure 8G). Moreover, Western blot was used to examined the key cell apoptosis regulators and found that miR‐139‐5p/miR‐940/miR‐193a‐5p mimic enhanced the protein expression of the active forms of caspase 3, caspase 7 and caspase 8 in the tumour tissues (Figure 8H). Therefore, the results indicated that miR‐139‐5p, miR‐940 and miR‐193a‐5p inhibited tumour growth in vivo through down‐regulating the expression of *SPOCK1*.

## 4 DISCUSSION

Down‐regulation of miR‐139‐5p, miR‐940 and miR‐193a‐5p was identified in HCC, and the inhibitory influence of these miRNAs imposed on HCC progression was revealed in this study. *SPOCK1, *the common target gene of miR‐139‐5p, miR940 and miR‐193a‐5p, enhanced HCC cell viability and invasion. Therefore, the regulatory effect of miR‐139‐5p, miR‐940 and miR‐193a‐5p on down‐regulating *SPOCK1* expression resulted in the suppression of HCC.

Down‐regulation of miR‐139‐5p, miR‐940 and miR‐193a‐5p was observed in HCC. Overexpression of these miRNAs in HCC cells could distinctively suppress proliferation and invasion, induce apoptosis and inhibit tumour growth. MiR‐139 down‐regulation was common in HCC. Its inhibitory effect on HCC cell proliferation and invasion was frequently found.^7^ According to previous studies, miR‐139‐5p restrained HCC deterioration through targeting several genes, such as *TCF‐4*,^7^ *ZEB1 *and *ZEB2*,^8^ *XIST* 1 and *c‐Fos*.^21^ By contrast, miR‐940's expression and effect in HCC have not been extensively studied. Ding et  al^12^ and Yuan et  al^11^ found out that the associations between miR‐940 expression and clinical features of HCC that lower the level of miR‐940 showed higher tumour grade, advanced invasion and reduced survival rate. As for miR‐193a, it was identified as a serum miRNA which was related to HCC.^22^ Down‐regulation of miR‐193a enhanced the proliferation inhibition induced by sorafenib, thus sensitizing the HCC cells to sorafenib therapy.^13^ MiR‐193a‐3p dictated the deterioration of HCC‐clinical analysis^10^ and reduced the 5‐FU resistance of HCC cells by mediating *SRSF2* *.* 23 Similarly, miR‐193b also facilitated sorafenib and cisplatin induced apoptosis,^14^, ^24^ indicating that miR‐193 could improve chemosensitivity. In summary, the antitumour effect of miR‐139‐5p has been reported in many researches. Our study confirmed its inhibitory effect on HCC. Limited studies in the relationship between miR‐940 and HCC focused on clinical and prognosis analysis. Our study, in detail, investigated the biological functions of miR‐940 in HCC cells, revealing the underlying reasons for miR‐940's predictive role. MiR‐193a‐5p's suppressive effect in HCC was reported for the first time here. However, miR‐193a‐3p and miR‐193b were all proved to exert antitumour effect in HCC, which was in line with miR‐193a‐5p, further verifying the anti‐carcinoma effect of miR‐193 family.

After inspecting miR‐139‐5p, miR‐940 and miR‐193a‐5p, *SPOCK1* was found out as their common target gene. Since *SPOCK1* was a tumour promotor that benefited HCC cell proliferation and invasion and reduced apoptosis, miRNAs exerted their inhibitory effect on HCC through down‐regulating *SPOCK1*. The tumour promotive effect in HCC was reported by Li et  al, whose study showed that *SPOCK1* could inhibit apoptosis and promote cancer invasion.^16^ Meanwhile, *SPOCK1* also played promotive roles in the progression of other cancers,^25^ so that it was considered as a novel metastasis biomarker and potential target.^26^ Many studies showed that *SPOCK1* could activate PI3K/AKT signalling pathway, leading to the promotion of gliomas,^27^ colorectal cancer^18^ and gall bladder cancer.^28^ In gastric cancer, miR‐129‐5p could inhibit the expression of *SPOCK1*, leading to the suppression of cancer cell processes.^20^ Nonetheless, the target relationship between *SPOCK1* and three miRNAs has not been properly investigated before. We found that *SPOCK1* could bind miR‐139‐5p, miR‐940 and miR‐193a‐5p, and it was suppressed by these miRNAs directly and specifically in HCC cells. This finding might be one of the potential mechanisms in inhibiting HCC development. As a common target gene of these miRNAs, *SPOCK1* played a vital role in stimulating HCC progression and had great potential to be a novel target for HCC treatment.

However, there still existed several limitations in our study. The sample size in our study is small and the relationships among these miRNAs need further exploration. In addition, we found that cotransfection of these miRNAs can further suppress the progression of HCC, while its effect on the inhibition of *SPOCK1* has no significant difference compared with single transfection. We suppose that these miRNAs have other target mRNAs which are tumour promoter and overexpression of these miRNAs inhibits these mRNAs the expression of these mRNAs leading to a better antitumour effect. However, its potential mechanism remains to be further investigated and confirmed. Since many previous studies report the involvement of *SPOCK1* in PI3K/AKT signalling pathway, further researches are worth conducting to investigate the signalling pathways regulated by *SPOCK1* in HCC and to identify their associations with miR‐139‐5p, miR‐940 and miR‐193a‐5p.

In conclusion, miR‐139‐5p, miR‐940 and miR‐193a‐5p were down‐regulated in HCC and their overexpression suppressed cell proliferation and invasion, induced apoptosis and inhibited tumour growth in vivo. *SPOCK1*, the common target gene of these miRNAs, was overexpressed in HCC and promoted its development. Therefore, through down‐regulating *SPOCK1*, miR‐139‐5p, miR‐940 and miR‐193a‐5p successfully restrained HCC deterioration.

## ETHICS APPROVAL AND CONSENT TO PARTICIPATE

This study was approved by the Human Research Ethics Committee of the First Affiliated Hospital of Guangzhou University of Chinese Medicine. Moreover, the experiments were undertaken with the understanding and written consent of each subject.

## CONFLICT OF INTEREST

The authors confirm that there are no conflicts of interest.

## AUTHOR CONTRIBUTIONS

P.L. substantially contributed to the concept and design of the work; P.L. and Y.Z involved in the analysis and interpretation of the data.; P.L., J.L. and Z.X. drafted the manuscript; L.L. critically revised the work for important intellectual content; all authors involved in the final approval of the work.
